# Neomycin, polymyxin B, and dexamethasone allergic reactions following periocular surgery

**DOI:** 10.1186/s12348-017-0133-4

**Published:** 2017-06-12

**Authors:** Nicholas A. Moore, Craig N. Czyz, Tracy D. Carter, Jill A. Foster, Kenneth V. Cahill

**Affiliations:** 1 Department of Ophthalmology, Indiana University School of Medicine, 2022 Ruckle St., Indianapolis, IN 46202 USA; 2 Division of Ophthalmology, Oculofacial Plastic and Reconstructive Surgery, Doctors Hospital, Columbus, OH USA; 3 Department of Ophthalmology, Oral and Maxillofacial Surgery, Grant Medical Center, Columbus, OH USA; 4 Ophthalmic Surgeons and Consultants of Ohio, Columbus, OH USA

## Abstract

**Background:**

The aim of this study was to evaluate the rate of periocular allergic skin reactions to topical neomycin, polymyxin B, and dexamethasone (NPD) ophthalmic ointment.

**Methods:**

A consecutive patient prospective study was performed. A total of 522 patients who had a procedure involving incision of the periocular skin with subsequent postoperative application of NPD ophthalmic ointment were included. Patients were evaluated for signs of allergy at 1 week postoperatively or prior if the patient had complaints. A periocular allergic reaction was defined as any periocular skin pruritus, erythema, edematous papules, vesicles, or plaques at the site of ointment application beyond that of the typical postprocedure presentation. The patients continued to be monitored for 30 days postoperatively.

**Results:**

Of the 522 patients who completed the study, eight (1.5%) had a definitive periocular allergic contact dermatitis to the NPD ophthalmic ointment. Allergic presentation ranged from postoperative day 3 to 14.

**Conclusions:**

The rate of periocular allergic reactions to NPD ophthalmic ointment is significantly lower than reported in the literature for other topical preparations of neomycin and polymyxin B. The low rate of allergy in this study suggests that NPD ophthalmic ointment can safely be applied to the periocular skin with a very minimal risk of inciting an allergic reaction.

## Background

An ophthalmic topical antibiotic is typically prescribed as routine postoperative care for a variety of periocular and ocular procedures [1, 2]. A commonly used ointment consists of neomycin sulfate (3.5 mg, 1.2% concentration), polymyxin B sulfate (10,000 units, less than 0.1% concentration), and dexamethasone (0.1%). In addition, neomycin, polymyxin B, and dexamethasone (NPD) ophthalmic ointment contains methylparaben (0.05%) and propylparaben (0.01%), preservative agents that provide antimicrobial activity and prevent biodegradation of the compound. The ointment also contains white petrolatum and anhydrous liquid lanolin, a carrier vehicle and viscosity increasing agent used to enhance bioavailability [3]. Furthermore, some NPD formulations may contain mineral oil.

The use of NPD ointment is contraindicated if there is known hypersensitivity to any component of the compound [3]. Each of the agents in this combined steroid/anti-infective ophthalmic ointment has been individually investigated for the incidence of topical allergic contact dermatitis—a type IV hypersensitivity reaction [4, 5]. Polymyxin B has a low sensitization rate, and there is not sufficient evidence to discourage its use because subsequent topical exposure to the antigen is unlikely to cause an allergic contact dermatitis [6–8]. Conversely, neomycin has been identified in the dermatologic literature as having high rates of sensitization due to previous antigen exposure, thereby increasing the susceptibility for an allergic contact dermatitis reaction with each successive contact. Estimates regarding neomycin allergy widely range in the literature [6–10]. For instance, the prevalence of allergic contact reaction to neomycin based on patch testing ranges from 1 to 6% [6–9]. Alternatively, Gehrig and Warshaw evaluated neomycin allergy rates from the North American Contact Dermatitis Group (NACDG) and estimated that 7–13% of patients were allergic to neomycin [10]. Several studies have concluded that because of the frequency of sensitization and risk of contact allergy upon re-exposure to the medication, neomycin-containing antibiotics should be avoided in postoperative open and closed wound care [11, 12]. These findings have potentially resulted in reluctance by surgeons to prescribe postprocedural topical neomycin and/or combination ointments containing this agent.

NPD ointment is preserved with methylparaben (0.05%) and propylparaben (0.01%). Parabens can trigger irritation and allergic hypersensitivity reactions, but the concentrations required (5–12%) are significantly higher than what is used in clinical practice [4]. Few studies examining parabens and ocular allergy are available in the literature. The latest patch test results from the NACDG revealed an allergy rate of 1.2% in the general population at a paraben concentration of 12% [6]. In addition, dexamethasone has been associated with a very low rate of allergic contact skin reactions [5]. While there are many different components within ophthalmic ointments that patients may have an allergic contact response to, most reactions appear to be secondary to chemical irritation. Only 10% of reactions to all topical ophthalmic medications are estimated to be the result of a true allergic response [13, 14].

The incidence of allergic reaction to NPD or any other steroid/anti-infective combination applied to the periocular surface has not been well documented, and none have investigated the postoperative use on open or closed wounds. A report in 1976 showed the incidence of periocular sensitivity reactions from a steroid/anti-infective combination to account for one case per million units (tube or bottle) of medication dispensed. Additionally, they reported that the statistical incidence of adverse reactions was no greater with the steroid/anti-infective combination compared with the separate components [15]. This and other reported studies are limited by retrospective analysis. In addition, most studies to date have been performed on the trunk/extremity and often involve a compromised cellular status such as seen in stasis dermatitis, eczema, or leg ulcerations. Further complicating the evaluation of true incidence is the fact that studies perform patch testing with a concentration far exceeding the levels found in ophthalmic ointments. The purpose of this study was to prospectively monitor the rate of periocular allergic contact reactions to topical NPD ointment following periocular surgical procedures in an attempt to elucidate an accurate allergy rate.

## Methods

The study was approved by the Mount Carmel Institutional Review Board (IRB)/Ethics Committee prior to data collection. HIPPA compliance was maintained, and the study adhered to the Declaration of Helsinki. Consecutive patients who had a procedure involving incision of the periocular skin with subsequent postoperative application of NPD ophthalmic ointment three times per day were included in the study. Exclusion criteria included age less than 18 years old; a reported history of allergy to neomycin, polymyxin B, and compounds containing them; or usage of intra- or postoperative systemic steroids. The physician completed a data collection sheet for all patients at the 7-day postoperative follow-up examination or prior if the patient presented for evaluation. Data recorded included patient age, sex, race, follow-up day, procedures performed, allergic reactions to the ointment, use of intra- or postoperative systemic steroids, and administration of postoperative oral antibiotics. An allergic reaction was defined as any periocular skin pruritus, erythema, edematous papules, vesicles, or plaques at the site of ointment application beyond that of the typical postprocedure presentation [10]. The patients continued to be monitored for 30 days postoperative via self-surveillance with physician evaluation if a suspected reaction was present. Patch testing was not conducted in this study to confirm allergic reactions to the NPD ointment.

Statistical analysis was performed using Prism v6.0 (Graphpad Software, Inc, La Jolla, CA). Analysis was conducted at the 0.05 alpha level with two-tailed *p* values. Fischer’s exact test was utilized to compare allergy rates between males and females and those receiving oral antibiotics. An unpaired *t* test was used to compare age of those with and without an allergic response. There was no indication for the use of multiple comparison correction. A priori power testing indicated a 95% power using the above parameters and a 5% allergy rate at 140 patients. Post hoc power analysis indicated the study achieved a power of 99%.

## Results

**Fig. 1:**
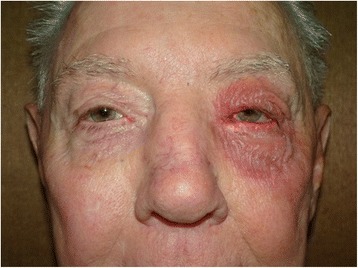
Patient on postoperative day 7 following left ectropion repair. The patient displays an allergic reaction to the NPD ointment that he applied two to three times a day to the upper and lower eyelid on the left

**Fig. 2:**
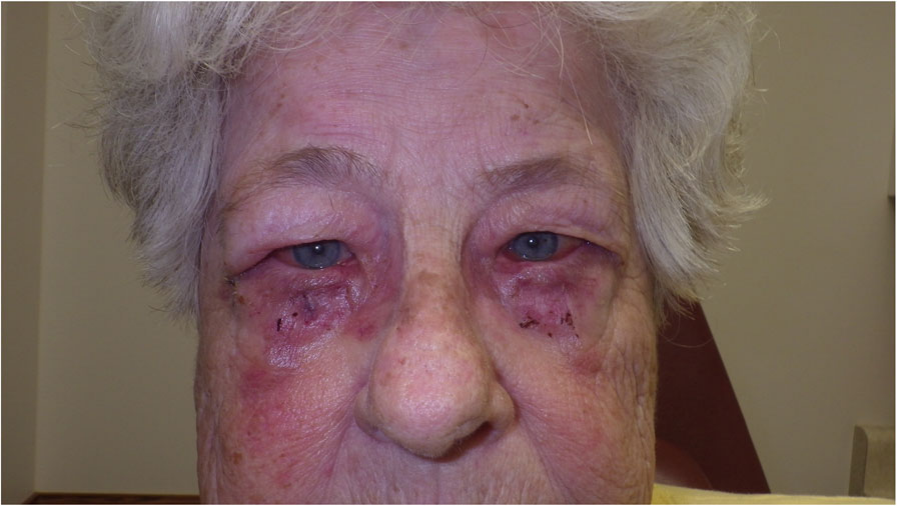
Patient on postoperative day 7 following bilateral lower eyelid entropion repair. This patient illustrates an allergic reaction to the NPD ointment that she applied two to three times a day. This particular patient was also instructed to apply ointment onto the ocular surface for dryness causing a conjunctival reaction to the NPD that can be seen in the form of conjunctival edema and hyperemia

A total of 522 patients (female—329 (63%) (average age 64 ± 18 years)/male—193 (37%) (average age 56 ± 19 years)) with an average age of 61 years (range—14–99 years, standard deviation ±19 years) were prospectively monitored after application of NPD ointment. Of these patients, eight (1.5%) patients (one male (13%), seven females (87%); average age 57 ± 20 years, range 13–74 years) were documented as having a periocular allergic reaction (Figs. 1 and 2). There was no significant statistical difference between male and female allergy rates (*p* = 0.268) or age of responders (*p* = 0.555). Allergic response ranged from postoperative day 3 to 14. One hundred eighty-seven patients (36%) were placed on systemic oral antibiotics for 1 week following their procedure. There was no significant statistical difference in incidence of allergic reaction between those who did and did not receive oral antibiotics (*p* = 0.142)

Five of the patients (63%) who experienced an allergic reaction had been prescribed postoperative oral antibiotics with no previous history of allergy or systemic reaction. Seven of the eight patients’ (88%) allergic symptoms improved once application of the NPD ointment was discontinued. One patient required administration of an oral steroid regimen to alleviate residual allergic inflammation.

There were two patients who had indeterminate periocular signs and symptoms of allergy at postoperative evaluation and were not included in the reaction group. One patient had swelling and itching, which was attributed to an allergic rhinosinusitis. The other patient presented with redness and swelling that was deemed to be a part of the expected postoperative response to surgery. Both patients were continued on NPD ointment with eventual resolution of their symptoms.

## Discussion

In this study population, 1.5% of patients with postoperative application of topical NPD ointment experienced a periocular allergic contact skin reaction. Despite the high rates of sensitization and allergy to neomycin reported in the literature, the results confirmed our anecdotal observation of low allergy rates with NPD ophthalmic ointment. In the study by Gehrig and Warshaw, it is estimated that up to 13% of patients are allergic to neomycin and at risk of experiencing an allergic contact dermatitis [10]. However, reports of topical allergic contact dermatitis from neomycin applied in postoperative surgical patients revealed a lower rate ranging from 4 to 5.3%, which more closely approximates the periocular allergic rates observed in this study [11, 12, 16]. Based on the results of this study compared with the risk estimates from previous reports, NPD ointment appears to have a lower risk of causing allergic contact dermatitis compared to other topical neomycin preparations.

In this study, there was no statistically significant age or gender difference in allergy rates (*p* = 0.555 and 0.268, respectively). de Pádua et al. conducted a retrospective multifactorial analysis and concluded that patients older than 60 years of age were 1.5 times more likely to have a neomycin allergy [9, 17]. The average age of eight patients who experienced a periocular skin reaction in our study was lower than those who had no reaction (57 versus 61 years). Furthermore, Nethercott et al. reported a higher proportion of positive patch test results to neomycin sulfate among females (*p* < 0.05) [18, 19]. In concordance with our study population, seven of the eight patients who experienced a contact dermatitis to NPD ointment were female; however, this was not a statistically significant difference. Thus, NPD ointment does not seem to have the same age- or gender-dependent risk factors when compared to neomycin alone.

The non-antibiotic components of NPD ointment have been shown to cause allergic hypersensitivity reactions, but as previously discussed, the concentrations required for this are 100 to 1000-fold greater to what is found in the NPD compound studied [4, 6]. Similarly, dexamethasone has been associated with a very low rate of allergic contact skin reactions [5]. While no conclusions can be made regarding the reactivity of individual components in NPD ointment, these findings support the low rate of allergic contact reactions observed in this study.

We recognize that this study is not without limitations; however, the prospective nature serves to minimize them. This study is at risk for observer bias as each of the participating physicians was aware that patients received NPD ointment. This may have influenced the decision to record an observed response as a normal postoperative reaction secondary to surgery as opposed to an allergic reaction to the ointment. A standardized data collection sheet was created in attempt to obtain a uniformed assessment from each physician to limit this bias. We also recognize that many dermatologic studies evaluated each entity of the NPD ointment individually. There is the potential that our low rate of periocular reactions could be secondary to a steroid suppression effect from the dexamethasone within the ointment—though one study reported no difference in allergic reactions between steroid/anti-infective combinations and each component individually [15]. Patch testing was not performed on any of the patients diagnosed with an allergic reaction to the NPD as the clinical evidence was sufficient to make the diagnosis. Due to the large sample size, we would expect patch testing to cause a false elevation of the clinically identifiable allergy reactions, not a reduced incidence.

## Conclusions

Based on the results of this prospective study, postoperative application of NPD ointment to the periocular skin has a low risk of causing an allergic reaction in patients with no history of allergy. Surgeons should not avoid NPD ointment in the postoperative setting for periocular surgery based upon the low allergic rate demonstrated. Future studies are needed to evaluate the periocular reactions experienced with application of individual topical antibiotics and other steroid/anti-infective combination agents.
